# Analysis of Dust Emission Characteristics of Peanut Whole-Feed Harvesting Based on Total Amount Collection Method

**DOI:** 10.3390/ijerph192315937

**Published:** 2022-11-29

**Authors:** Hongbo Xu, Peng Zhang, Zhichao Hu, Enrong Mao, Zhaoyan You, Yuefeng Du

**Affiliations:** 1 College of Engineering, China Agricultural University, Beijing 100083, China; 2 Nanjing Institute of Agricultural Mechanization, Ministry of Agriculture and Rural Affairs, Nanjing 210014, China

**Keywords:** peanut, whole-feed harvest, dust, emission characteristics, total amount collection

## Abstract

In view of the lack of a total amount collection method of dust emitted from a peanut whole-feed harvester and the unknown characteristic parameters of dust emission, a total amount dust collection method based on the combined action of centrifugation and filtration was proposed. The structural parameters of a total amount dust collection device were designed through theoretical analysis. On this basis, the production of a total amount dust collection device and a total amount dust collection test of the peanut whole-feed harvester were completed. The test showed that the total amount dust collection device could meet the needs of dust emission characteristics research. After analyzing the collected dust, it was found that the emitted particles were a mixture of soil particles and fiber particles. When the engine speed of the harvester was increased from 1600 rpm to 2400 rpm, the total emission rate increased from 3.36% to 4.28%, and the particulate emission rate increased from 1.44% to 2.63%; it also caused 4.29~4.98% of seedling blowing loss. The emission proportion of soil particles was reduced from 58.93% to 44.25%, and the emission proportion of fiber particles was increased from 41.07% to 55.75%. Among the emitted particles, the particle size peak of soil particles was concentrated at 22.9~30.2 μm; the particle size peak of fiber particles was concentrated at 478.6~631.0 μm. The research method and results can provide a reference for the optimization of dust reduction and emission reduction of peanut whole-feed harvesters and similar crop harvesters.

## 1. Introduction

Peanut whole-feed harvesting, which comprises a series of operations such as pick-up, picking, cleaning, and collecting at the same time, has the advantages of low operating cost and high efficiency, and is the most popular mechanized peanut harvesting mode all over the world [1]. With the continuous innovation and progress of the mechanized peanut harvesting technology, the peanut whole-feed harvester has been widely used in the main production areas of China [2,3]. However, a large amount of dust is emitted from the harvester under the action of the beating, kneading, and cleaning of the whole-feed peanut plants and the entrained soil [4]. Dust exposure in and near farms is of increasing concern because of the damage it inflicts on human respiratory systems [5]. The widely existing free silica dust can lead to silicosis. At the same time, the large amount of dust around the machines has adverse effects on the work quality and performance of the machines [6,7,8].

With the deepening of the construction of ecological civilization and the concept of green agricultural development, the research and development of green mechanization technology has become an important direction in the development of agricultural mechanization in China and even the world [9]. The improvement of dust suppression of peanut whole-feed harvesting has attracted the attention of management departments and academia [10]. However, at present, it is proposed to install sprinklers on harvesters for dust suppression during harvesting operations, but the feasibility of agricultural production has not been considered in depth, which has been criticized and questioned by the masses and mainstream media [11]. Therefore, the problem of dust pollution from peanut whole-feed harvesting operations has not been effectively solved, among which an important factor is that the characteristic parameters of dust emitted by the harvester are still unknown, and the research on dust suppression lacks detection and evaluation means.

Dust collection is the basis of dust characteristic analysis. For the collection method of dust emitted in agricultural production, numerous scholars have produced a large amount of research [12,13,14]. Xu et al. [15] sampled dust discharged from a peanut whole-feed harvester using an integrated atmospheric sampler that enabled laboratory measurements of dust concentration, particle size distribution, density, and shape factors. Foqué et al. [16,17] used a mechanical vibrating screen to separate dust particles abraded from pesticide-treated seeds and tested the particle size distribution, envelope density, porosity, and chemical content of the dust particles. Zhang et al. [18] collected the dust discharged from a rice wheat combine harvester with an air suction dust collector and measured the composition and particle size distribution of the dust. Tian et al. [19] used a dust collection filter bag to collect the materials under the axial threshing drum of rape and screened the threshing dust samples with a standard sieve. It can be seen from the above research that current studies generally adopt a sampling method for dust collection of agricultural machinery operations, but due to differences in sampling location, suspension characteristics of dust components, dust particle size distribution, etc., the sampling method cannot fully represent the characteristics of dust emitted from agricultural machines, which affects the accuracy of dust hazard assessment and emission mechanism research.

In view of the above problems, this study proposed a total amount dust collection method based on the combined action of centrifugation and filtration according to the typical peanut whole-feed harvesting technology model, developed a total amount dust collection device, and conducted collection tests. The relationship between the dust emission and working parameters of a typical peanut whole-feed harvester was obtained, and the dust composition and particle size distribution characteristics were detected. This study provided a new method and data support for further evaluation of dust pollution and dust suppression technology of harvesters.

## 2. Materials and Methods

### 2.1. Methods of Total Amount Dust Collection

#### 2.1.1. Analysis of the Demand for Total Amount Dust Collection

**Figure 1:**
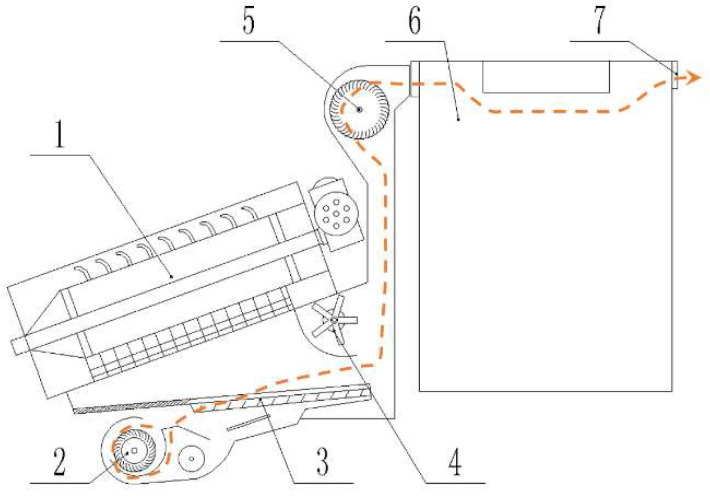
Schematic of dust emission flow of peanut whole-feed harvester: 1. Threshing cylinder. 2. Cleaning fan. 3. Cleaning screen. 4. Stalk crushing blade. 5. Stalk lifting fan. 6. Stalk collecting box. 7. Dust outlet. (Note: Orange dotted line indicates the direction of dust exhaust airflow).

In this paper, the goal was to collect the total amount of dust emitted by a peanut whole-feed harvester (CF326). This harvester was a mainstream type of peanut whole-feed harvester in the market, with good representativeness. Figure 1 is a schematic diagram of the dust emission flow of this peanut whole-feed harvester. It can be seen that the dust emission flow passed through the working parts, including the cleaning fan, stalk lifting fan, and stalk collecting box, etc. During operation, after the separation of peanut pods and stalks by the threshing cylinder, the whole stalks rotated with the threshing cylinder and were transported to the stalk crushing blade. Peanut pods and light impurities entered the cleaning screen for secondary separation. The crushed whole stalks and light impurities entered the dust emission flow together and formed a mixture of crushed stalks and particles. The mixture of crushed stalks and particles moved to the cover plate of the stalk collecting box and was dispersed. The crushed stalks with high suspension speed settled in the stalk collecting box and were used for feed processing, while the dust particles with smaller suspension velocity left the stalk collecting box and were discharged into the atmosphere with the dust emission flow.

Affected by the structure of the straw collecting box, the wind speed distribution at the dust outlet is uneven. The measurement results show that the wind speed at the sides of the dust outlet is more than twice that in the middle. Therefore, a wind speed matrix method was used to measure the dust emission flow of the harvester at the dust outlet. The dust outlet of the harvester was evenly divided into *n* sampling areas (*n* = 10), and the wind speeds at each central point were measured with an impeller anemometer (Testo 417); then, the dust discharge flow of the harvester is:

(1) $$Q_{H}=3600\times \overset{10}{\underset{n=1}{\sum }}v_{n}\cdot S_{n}$$

where *Q_H_* is the dust emission air volume of the harvester, m^3^/h; *v_n_* is the wind speed at the center of the *n* sampling area, m/s; *S_n_* is the area of the *n* sampling area, m^2^. According to the product manual and the basis of previous research, the normal working speed range of the engine of the peanut whole-feed harvester is 1600~2400 rpm; therefore, the measured and calculated dust emission air volume range of the harvester was 6880~10,320 m^3^/h.

#### 2.1.2. Device of Total Amount Dust Collection

**Figure 2:**
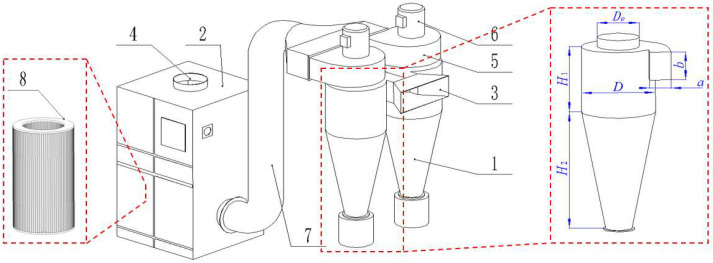
Total amount dust collection device: 1. Cyclone separator. 2. Filter cartridge dust collector. 3. Air flow inlet. 4. Air flow outlet. 5. Cyclone separator fan. 6. Variable frequency motor of cyclone separator. 7. Pipeline. 8. Filter element.

According to the dust emission mode and exhaust air volume range of the peanut whole-feed harvester, this paper proposed a device for total amount collection based on the combined action of centrifugation and filtration. As shown in Figure 2, the technical scheme of cyclone separators in series with a filter cartridge dust collector was used to achieve the goal of total amount dust collection.

A cyclone separator is a kind of common equipment used for separating a gas–solid system. Its working principle is that the solid particles with large inertial centrifugal force are thrown to the external wall surface by the rotary movement caused by the tangential introduction of air flow, and they settle to the bottom of the cyclone separator under the action of friction and gravity [20]. It has the advantages of having a compact structure, high efficiency, and being low-cost, and it is commonly used to capture particles with a diameter of more than 5~10 μm [21]. The capture efficiency is usually greater than 90% within the working parameter range. The size of the cyclone separator increases with the increase in its gas flow rate, while the separation efficiency decreases [22]. Therefore, two counter-rotating cyclones with smaller diameters were used in parallel to form a cyclone separator group.

The filter cartridge dust collector is composed of an air inlet pipe, exhaust pipe, box, dust hopper, dust cleaning device, diversion device, fan, filter element, and electric control device. After the dusty gas enters the dust collector box, due to the sudden expansion of the air flow section and the effect of the air flow distribution plate, some coarse particles in the air flow settle in the dust hopper under the action of dynamic and inertial forces. After the dust particles with a fine particle size and low density enter the dust filtering chamber, the dust is deposited on the surface of the filter material through the combined effect of Brownian diffusion and screening, and the purified gas enters the air purification chamber and is discharged by the exhaust pipe through the fan. When the thickness of the dust layer on the filter material surface reaches a specified value, the pulse valve opens automatically for dust removal. As the filter material is folded into a cylinder for use, the arrangement density of the filter material is large, so the dust collector is compact in structure and small in volume. With the development of new technology and new materials, the capture and recovery efficiency of filter cartridge dust collectors for more than 1 μm dust can reach more than 99.6% [23].

During dust collecting, the dust particles with a larger particle size and the blown crushed stalks were easily captured by the cyclone separator after the dusty air flow entered the cyclone separator group, while the dust particles with a smaller particle size escaped from the outlet of the cyclone separator group and entered the filter cartridge dust collector for secondary capture. This collection method not only effectively used the capture characteristics of the cyclone separator and the filter cartridge dust collector, but also avoided the blockage and damage of the filter element and prolonged the service life of the equipment. The fans of the cyclone separator and the filter cartridge dust collector were driven by variable frequency motors, which could match the dust discharge air volume of the harvester during operation, so as to avoid the influence of the collection device on the original dust discharge flow field.

#### 2.1.3. Key Parameter Design

The inlet air speed of the cyclone separator under normal operation is 15~25 m/s, so when the air volume collection demand of the harvester is met, the inlet sectional area of a single cyclone separator is:

(2) $$\{\begin{array}{c} 15\times A\times 3600<\frac{Q_{min}}{n_{c}}\\ 25\times A\times 3600>\frac{Q_{max}}{n_{c}} \end{array}$$

where *A* is the inlet sectional area of a single cyclone separator, m^2^; *Q_min_* is the minimum collection air volume demand, which is 6880 m^3^/h; *Q_max_* is the maximum demand for air volume collected, which is 10,320 m^3^/h; and *n_c_* is the number of cyclone separators, which is 2. The entrance width and height can be calculated by the following formula [24]:

(3) $$\{\begin{array}{c} a=\sqrt{\frac{A}{1.5}}\\ b=\sqrt{1.5\times A} \end{array}$$

where *a* is the entrance width, m; *b* is the entrance height, m. The cylinder diameter of the cyclone separator is [24]:

(4) D=3.5×a

where *D* is the diameter of the cyclone separator cylinder, m.

The pressure drop of the cyclone separator can be expressed by the following formula:

(5) $$\Delta P=\xi \frac{u_{i}^{2}\rho }{2}$$

where ∆*P* is the pressure drop of the cyclone separator, N/m^2^; *u_i_* is the inlet wind speed, which is 15~25 m/s; *ρ* is the gas density, which is 1.225 kg/m^3^; and *ξ* is the resistance coefficient, which is determined by the following formula [25]:

(6) $$\xi =11.3{\left(\frac{A}{D_{e}^{2}}\right)}^{2}+3.33$$

where *D_e_* is the diameter of the exhaust pipe, m, which can be taken as

(7) $$D_{e}=0.58\times D$$

For other dimensional relationships, refer to [24].

The filter element of the filter cartridge dust collector is a high-density polyester fiber with an outer diameter of 320 mm, an inner diameter of 220 mm, and a height of 660 mm. The number of filter elements is calculated by:

(8) $$n_{f}\geq \frac{Q_{max}}{3600\times S_{f}\times v_{f}}$$

where *n_f_* is the number of filter elements; *Q_max_* is the maximum demand for air volume collected, which is 10,320 m^3^/h; *S_f_* is the filtering area of a single filter element, which is 9 m^2^; and *V_f_* is the filtering wind speed, which is 0.036 m/s [26].

The key parameters of the total dust collection device are shown in Table 1.

### 2.2. Test of Total Amount Dust Collection

**Figure 3:**
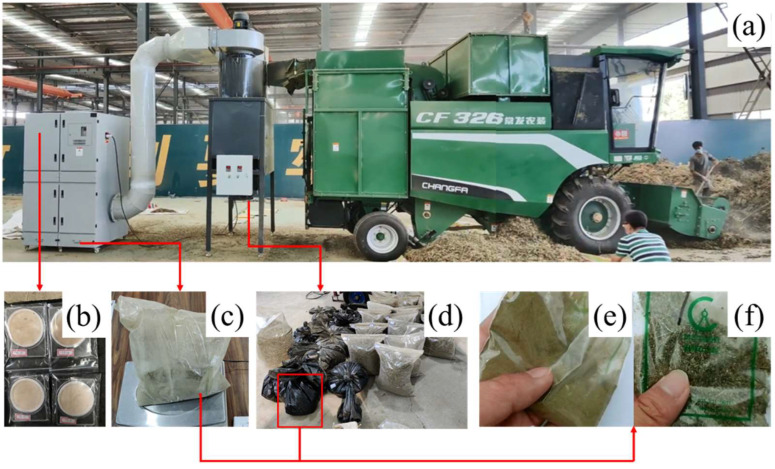
Total amount collection test of dust discharged from peanut whole-feed harvester: (**a**) Total amount collection test; (**b**) Dust sample at air flow outlet on filter membrane; (**c**) Dust sample collected by filter cartridge dust collector; (**d**) Dust sample (screened into coarse dust and broken stalks) collected by cyclone separator; (**e**) Soil particles from coarse dust; (**f**) Fiber particles from coarse dust.

In September 2021, a total amount collection test of dust emitted from a peanut whole-feed harvester was carried out in Suiping County, Zhumadian City, Henan Province (as shown in Figure 3a). The peanut variety used in the test was No. 2 Wanhua. The fed materials (including pods, stalks, and entrained soil) were sampled, and the moisture content (dry basis) of each component of the material was detected using a drying box (105 °C drying method) and electronic balance. The results show that the proportion of pods was 44.63%, with a moisture content of 16.38%, the proportion of stalks was 38.49%, with a moisture content of 19.81%, and the proportion of soil was 16.88%, with a moisture content of 4.83%.

The test was carried out at three different engine speeds (1600 rpm, 2000 rpm, and 2400 rpm). The air volume collected by the total dust collection device was adjusted to be consistent with the air volume of dust emissions. After the harvester and the collection device were running stably, 250 kg of materials were fed manually for each test. The feed rates for each test were 2 kg/s, 2.5 kg/s, and 3 kg/s (error within ±10%). After the test, dust in the cyclone separator and in the filter cartridge dust collector were collected, respectively. A total of three groups of tests were conducted, and each test was repeated three times. Test settings are shown in Table 2.

During the collection test, the collection performance of the total dust collection device was verified. The medium flow sampler (KB-120F) was used to sample the dust at the fan outlet of the filter cartridge dust collector (Figure 3b), and the filter membrane weighing method [27] was used to calculate the dust concentration at the outlet, and the dust discharge flow could be calculated on this basis. The results show that the collection rate of the total dust collection device for the discharged dust from the peanut whole-feed harvester was 99.78%, of which the collection rate of the cyclone separator was 95.69%, and the collection rate of the filter cartridge dust collector was 4.09%. The measurement results prove that the total dust collection device was effective, which could meet the research requirements of the characteristics of discharged dust from peanut whole-feed harvesters.

In the materials collected by the cyclone separator, it was found that the dust particles were mixed with some complete crushed stalks and leaves, which was the loss of materials due to being blown out of the dust exhaust airflow. Therefore, the sampled dust is screened by a 50-mesh sieve (355 μm) to obtain coarse dust and broken stalks (Figure 3d). The coarse dust collected in each test and the fine dust collected in the filter cartridge dust collector (Figure 3c) were regarded as the total amount of dust after mixing.

In order to further analyze the composition of the total amount of dust, distilled water was used to soak the total amount of dust. The settled dust was soil particles (Figure 3e), and the floating dust was plant fiber particles (Figure 3f).

### 2.3. Test of Dust Emission Characteristics

#### 2.3.1. Composition of Dust Particles

According to the material composition, it was predicted that the total amount of dust contained soil particles and plant fiber particles, but the actual particle composition could not be observed with the naked eye. Therefore, a scanning electron microscope (EVO MA 10/LS 10) was used to observe the composition of the total amount of dust particles collected by the total amount dust collection device from a peanut whole-feed harvester.

#### 2.3.2. Dust Emission Rate

The dust emission rate of the peanut whole-feed harvester could be obtained by weighing the collected materials with an electronic balance. The calculation formula of the emission rate test index is as follows:

(9) $$\eta_{a}=\frac{m_{c}+m_{f}}{m_{0}}\times 100\%$$

where *η_a_* is the total emission rate, %; *m_c_* is the weight of materials collected by the cyclone separator, g; *m_f_* is the weight of materials collected by the filter cartridge dust collector, g; and *m*_0_ is the weight of the feeding material, g. The emission rate of crushed stalks is:

(10) $$\eta_{s}=\frac{m_{s}}{m_{0}}\times 100\%$$

where *η_s_* is the emission rate of crushed stalks, %; *m_s_* is the weight of crushed stalks screened from the materials collected by the cyclone separator, g; and *m*_0_ is the weight of the feeding material, g. The emission rate of particulate matter is:

(11) $$\eta_{p}=\frac{m_{c}+m_{f}-m_{s}}{m_{0}}\times 100\%$$

where *η_p_* is the particulate matter emission rate, %; *m_c_* is the weight of the materials collected by the cyclone separator, g; *m_f_* is the weight of the materials collected by the filter cartridge dust collector, g; *m_s_* is the weight of the crushed stalks screened from the materials collected by the cyclone separator, g; and *m*_0_ is the weight of the feeding material, g.

The weight ratio of the soil particles in the total amount of dust is:

(12) $$\varphi_{s}=\frac{m_{1}}{m_{c}+m_{f}-m_{s}}\times 100\%$$

where *φ_s_* is the weight ratio of soil particles in the total amount of dust particles, %; *m*_1_ is the weight of the soil particles, g; *m*_c_ is the weight of the materials collected by the cyclone separator, g; *m_f_* is the weight of the materials collected by the filter cartridge dust collector, g; and *m_s_* is the weight of the crushed stalks screened from the materials collected by the cyclone separator, g. The weight ratio of fiber particles is:

(13) $$\varphi_{b}=\frac{m_{2}}{m_{c}+m_{f}-m_{s}}\times 100\%$$

where *φ_b_* is the weight ratio of the fiber particles in the total amount of dust particles, %; *m*_2_ is the weight of the fiber particles, g; *m_c_* is the weight of the materials collected by the cyclone separator, g; *m_f_* is the weight of the materials collected by the filter cartridge dust collector, g; and *m_s_* is the weight of the crushed stalks screened from the materials collected by the cyclone separator, g.

#### 2.3.3. Size Distribution of Dust Particles

The size distribution of the dust particles is one of the most important characteristics of discharged dust particles from peanut whole-feed harvesters considered in this study. A laser particle size analyzer (Mastersizer 2000, accuracy: 0.01 mm) was used to measure the particle size distribution of the total amount of collected dust. This particle size analyzer makes an equivalent comparison based on the scattering characteristics of particles to laser. The measured equivalent particle size is the equivalent scattering particle size, that is, the diameter of the spherical particle with the same scattering effect as the actual particle is used to represent the size of the actual particle (usually irregular in shape).

## 3. Results

### 3.1. Composition of Dust Particles

**Figure 4:**
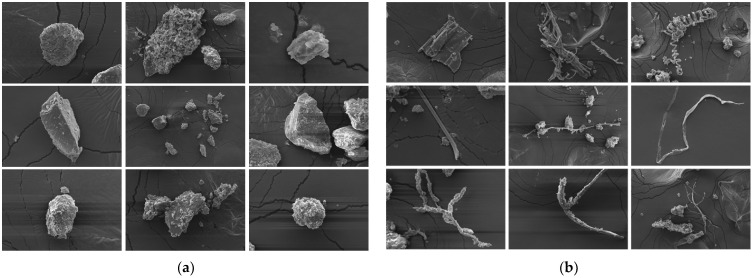
SEM images of the dust particles from the total amount of dust of a peanut whole-feed harvester: (**a**) Soil dust particles and (**b**) Fiber dust particles.

Due to the impact of human factors on the sampling method of the total amount of dust and the selection of the field of view of the electron microscope, these SEM images of dust particles cannot fully reflect the morphological characteristics of the total amount of dust particles, but they are enough to see that the total amount of dust was composed of soil particles (silica powder) and fiber particles. Panels (a) and (b) of Figure 4 are two representative images of the total amount of dust particles. The granular minerals in Figure 4a are the components of collected soil, and the fiber particles in Figure 4b were derived from the fiber of crushed peanut stalks and other plant fibers in the environment.

Dust particles with rough edges can irritate lung tissue and may induce interstitial lung fibrosis [28,29]. Particles with sizes of 2–10 μm float in the air for more than 100 h and can be inhaled [30]. The lightweight particles from crushed peanut stalks may float over long distances, affecting the air quality and the health of agricultural workers.

### 3.2. Dust Emission Rate

**Figure 5:**
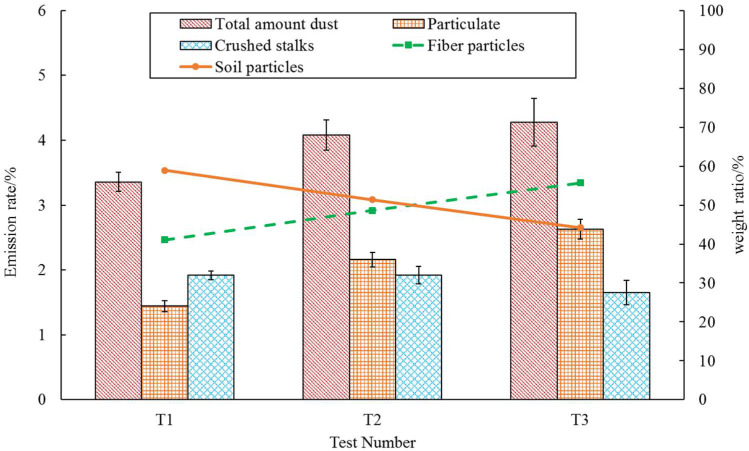
Emission statistics of a peanut whole-feed harvester under different air volumes. The error bars (black) are the standard deviations (SD). The histogram represents the emission rate (mean ± SD), while the line chart represents the weight ratio.

Figure 5 shows the dust emission rate of peanut whole-feed harvester under different air volumes. The small error bars show the good consistency of the new collection device and method. As shown in Figure 5, the total emission rate and particulate emission rate increased with the increase in the dust emission air volume. When the air volume increased from 6880 m^3^/h to 10,320 m^3^/h, the total emission rate increased from 3.36% to 4.28%, and the particulate emission rate increased from 1.44% to 2.63%. At the same time, 1.65~1.92% of the crushed stalks were discharged with the dust emission airflow, which could be regarded as the harvest loss of the stalks, accounting for 4.29~4.98% of the total peanut stalks.

It can be seen from Figure 5 that, with the increase in the dust emission air volume, the weight ratio of the soil particles decreased from 58.93% to 44.25%, and the weight ratio of the fiber particles increased from 41.07% to 55.75%. It can be seen that the growth rate of the fiber particle emissions was far higher than that of the soil particles.

### 3.3. Size Distribution of Dust Particles

**Figure 6:**
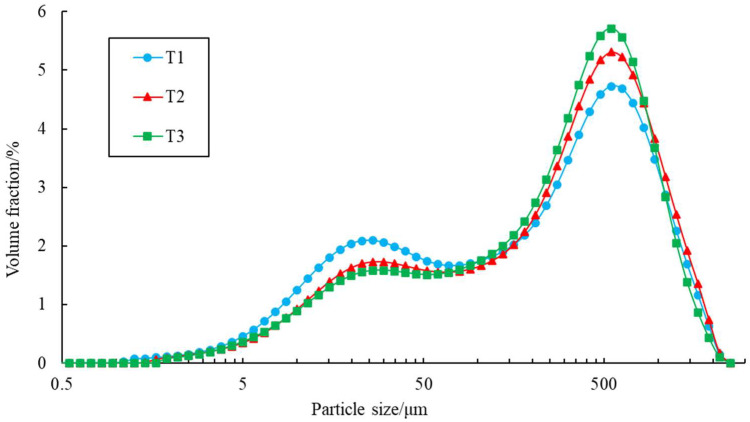
Particle size distribution of a peanut whole-feed harvester under different air volumes.

Figure 6 clearly shows that the particle size distribution had two peaks; this is because the collected dust particles were a mixture of soil particles and fiber particles. With the increase in the dust discharge air volume, the volume fraction of small particles decreased, while the volume fraction of large particles increased. From the analysis in Figure 6, it can be seen that the dust particles with a smaller particle size were soil particles, while those with a larger particle size were fiber particles, which is consistent with the change trend of soil particles and fiber particles in Figure 5. The peak particle size of the soil particles was around 22.9~30.2 μm, while the peak particle size of the fiber particles was around 478.6~631.0 μm.

## 4. Discussion

The total emission rate and particulate emission rate increased with the increase in the dust emission air volume. The reason for this may come from two aspects. On the one hand, more particles were formed due to the high rotating speed of working parts, and on the other hand, the carrying capacity of the air flow was enhanced due to the increase in air volume. With the increase in the dust emission air volume, the proportion of fiber particles in the total amount of dust increased. This may be due to the increase in the operating speed of the implement, resulting in more fibrous particles, or it may be that the fiber particles were more easily blown out by a high-speed flow field due to their low suspension speed compared with soil particles.

Peanut stalks are a high-quality livestock and poultry feed [31]. Their protein content is six times that of rice straw. About 5000 kg of peanut stalks can be obtained per hectare of peanut plants. However, when a peanut whole-feed harvester harvests peanut pods and stalks, the loss rate of stalks accounts for 4.29~4.98% of the total collected stalks. In actual production, due to the uncertainty of the operation process of the harvester, these losses may be more. Therefore, how to reduce the harvest loss rate of stalks is a new problem that needs to be paid attention in the process of machine optimization.

Early studies have found that the size constant of dust particles collected by an integrated atmospheric sampler from a peanut whole-feed harvester in the field was 23 μm [15], while in this study, the peak particle size of the soil particles was around 22.9~30.2 μm. The results of the two studies are basically consistent, but compared with outdoor collection, this study collected more larger fiber particles, so the particle size distribution results are larger than those in reference [14]. The measuring results of the fiber particles were rather large. The reason for this is that most fiber particles are long and irregular strips, while the laser particle sizer (Mastersizer 2000) calculates the particle size as an equivalent sphere direct (ESD), which deviates from the actual shape [32].

Due to the limitation of test costs, this paper only studied the emission rate, composition, and particle size distribution of the total amount of discharged dust from a peanut whole-feed harvester under three kinds of dust discharge flow rates. Further research can be carried out from two aspects. On the one hand, for the dust discharged from the harvester, more attention ought to be paid to the emission flow of PM_2.5_, PM_10_, and TSP under different working parameters. Therefore, the particle size distribution and true density can be detected for the separated soil and fiber particles, respectively, and then the emission flow of mixed particles under different particle sizes can be calculated according to the volume fraction and emission rate. On the other hand, as the rotation speed of all working parts of the harvester changes proportionally to the engine speed, it is impossible to accurately identify the factors affecting the dust emission characteristics and their influencing mechanisms. In subsequent research, a dust emission test platform will be built, and the rotation speed of the working parts of the harvester, such as the feeding device, the picking roller, the cleaning fan, the stalk crushing blade, and the stalk lifting fan, will be adjusted independently, to further explore the internal relationship between the operating parameters of the machine and the dust emission characteristics.

## 5. Conclusions

To study the characteristics of dust discharged from the peanut whole-feed harvester, a total amount dust collection method based on the combined effect of centrifugation and filtration was proposed. First, the large-size particles were collected through the cyclone separator, and then the escaped particles were captured twice by the filter cartridge dust collector. The test results show that the collection rate of the device could reach 99.78%, meeting the demand for the total amount of dust collection.

According to the analysis of the collected dust, it was found that the emitted particles from the peanut whole-feed harvester were a mixture of soil particles and fiber particles. When the engine speed of the harvester was increased from 1600 rpm to 2400 rpm, the total emission rate increased from 3.36% to 4.28%, and the particulate emission rate increased from 1.44% to 2.63%; it also caused a 4.29~4.98% seedling loss. The emission proportion of soil particles was reduced from 58.93% to 44.25%, and the emission proportion of fiber particles was increased from 41.07% to 55.75%. Among the emitted particles, the peak particle size of the soil particles was concentrated at 22.9~30.2 μm, while the peak particle size of the fiber particles was concentrated at 478.6~631.0 μm. The study method and results provide a reference for further evaluation of dust pollution and dust suppression technology of peanut whole-feed harvesters and similar crop harvesters.

## Figures and Tables

**Table 1:** Key parameters of total amount dust collection device.

Symbol	Index	Value	Sources
*A*	Inlet area of cyclone separator/m^2^	0.06	Calculated by Equation (2)
*a*	Inlet width of cyclone separator/m	0.2	Calculated by Equation (3)
*b*	Inlet height of cyclone separator/m	0.3	Calculated by Equation (3)
*D*	Diameter of cyclone separator cylinder/m	0.7	Calculated by Equation (4)
*D_e_*	Diameter of cyclone separator exhaust pipe/m	0.4	Calculated by Equation (7)
*H*_1_	Height of cyclone separator cylinder/m	0.7	Ref. [24]
*H*_2_	Cone height of cyclone separator/m	1.2	Ref. [24]
*ξ*	Resistance coefficient of cyclone separator	4.92	Calculated by Equation (6)
∆*P*	Pressure drop of cyclone separator/(N∙m^−2^)	675.1~1875.4	Calculated by Equation (5)
*n_f_*	Number of filter elements of filter cartridge dust collector	9	Calculated by Equation (8)

**Table 2:** Settings of test parameters.

Test No.	Engine Speed/rpm	Air Volume/(m^3^∙h^−1^)	Peanut Plants Weight/kg	Feed Quantity/(kg∙s^−1^)
T1	1600	6880	250	2 (±10%)
T2	2000	8600	250	2.5 (±10%)
T3	2400	10,320	250	3 (±10%)

## Data Availability

The datasets used and/or analyzed during the current study are available from the corresponding author on reasonable request.
